# The Role of Minimally Invasive Surgery in Appendectomy Within a Hernia

**DOI:** 10.7759/cureus.10630

**Published:** 2020-09-24

**Authors:** Anupam K Gupta, Oscar A Vazquez, Monica I Burgos, Jose Yeguez

**Affiliations:** 1 Minimally Invasive Surgery, University of Miami Hospital, Miami, USA; 2 Surgery, Florida Atlantic University Charles E. Schmidt College of Medicine, Miami, USA; 3 Anesthesiology/Internal Medicine, Universidad Autonoma de Guadalajara, Guadalajara, MEX

**Keywords:** hernia, appendectomy

## Abstract

We present three cases where an inflamed incarcerated appendix was in a femoral, inguinal, and an umbilical hernia. All three patients underwent an appendectomy laparoscopically. The hernias in two of the patients (femoral and inguinal) were left unrepaired as the primary goal was to relieve the patients' symptoms and achieve source control. The hernia was repaired primarily in the patient with an umbilical hernia intraoperatively. At three months follow-up, none of the patients had a clinically visible hernia.

## Introduction

A hernia is a projection of an organ, or a part of an organ, through the body wall that usually contains it. De Garengeot's hernia is a rare subtype of an incarcerated femoral hernia, which describes the incarceration of the vermiform appendix within a femoral hernia [1]. Incarceration of the appendix within an inguinal hernia is Amyand's hernia, and it can become inflamed, infected, or perforated, or be incarcerated and completely healthy [2]. An umbilical hernia containing an appendix has no eponym for it yet. Our literature search revealed only 11 available case reports describing an umbilical hernia containing an inflamed appendix [3]. While most authors believe in an open approach to incarcerated hernias, we describe the benefits of laparoscopy in this subset of patients.

## Case presentation

Case one 

**Figure 1:**
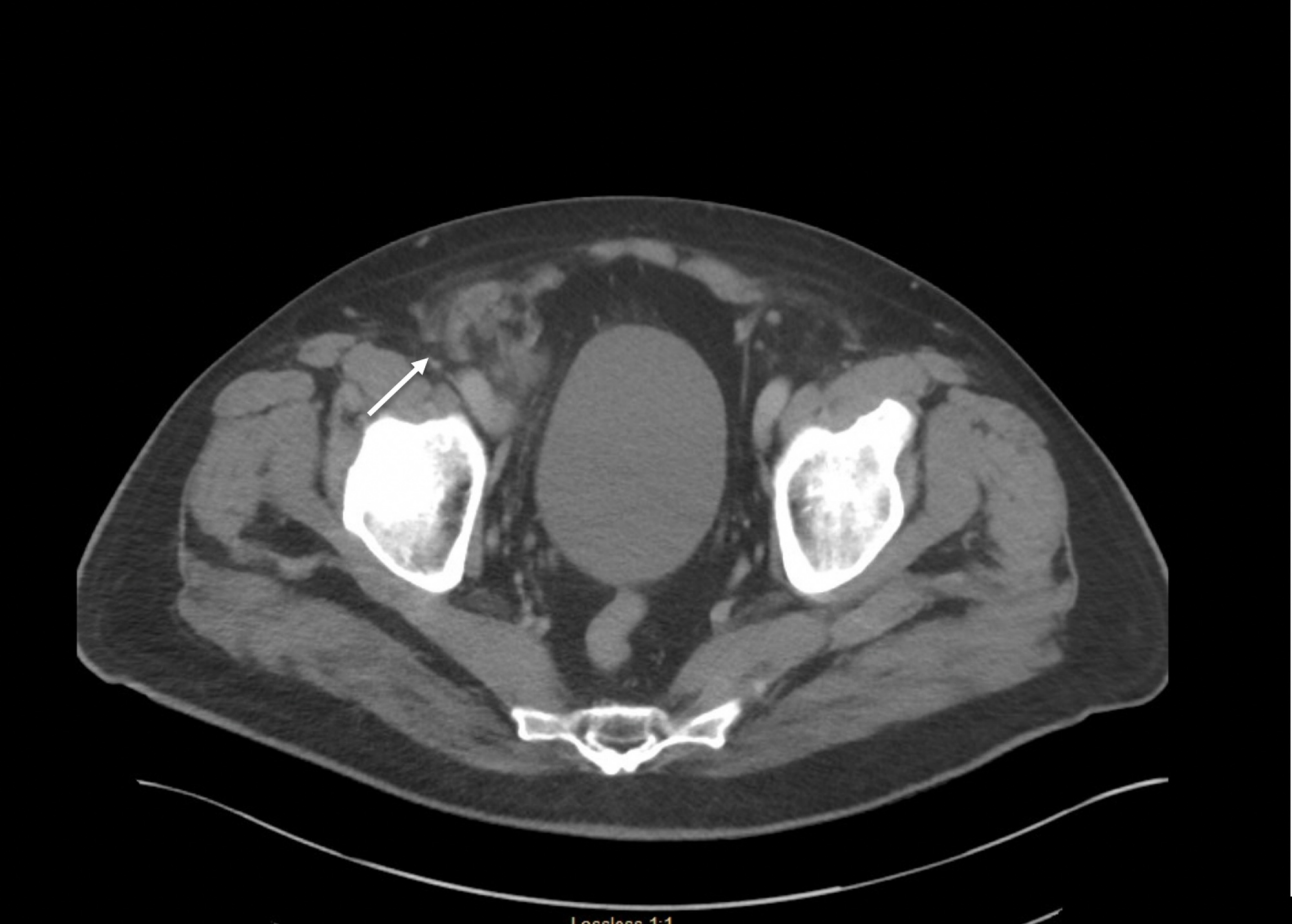
CT scan of the abdomen and pelvis revealing an incarcerated right femoral hernia with an inflamed appendix CT = computed tomography

A 68-year-old woman with a past medical history of hypertension with prior hysterectomy presented with acute pain over her right groin. On arrival to the emergency room, her vital signs were stable, and clinical examination was remarkable only for pain and tenderness at the right groin on palpation. Her blood work was significant for leukocytosis of 20x103 WBC/mL. A computed tomography (CT) of the abdomen and pelvis revealed an incarcerated right femoral hernia with an inflamed appendix as its contents (Figure 1).

**Figure 2:**
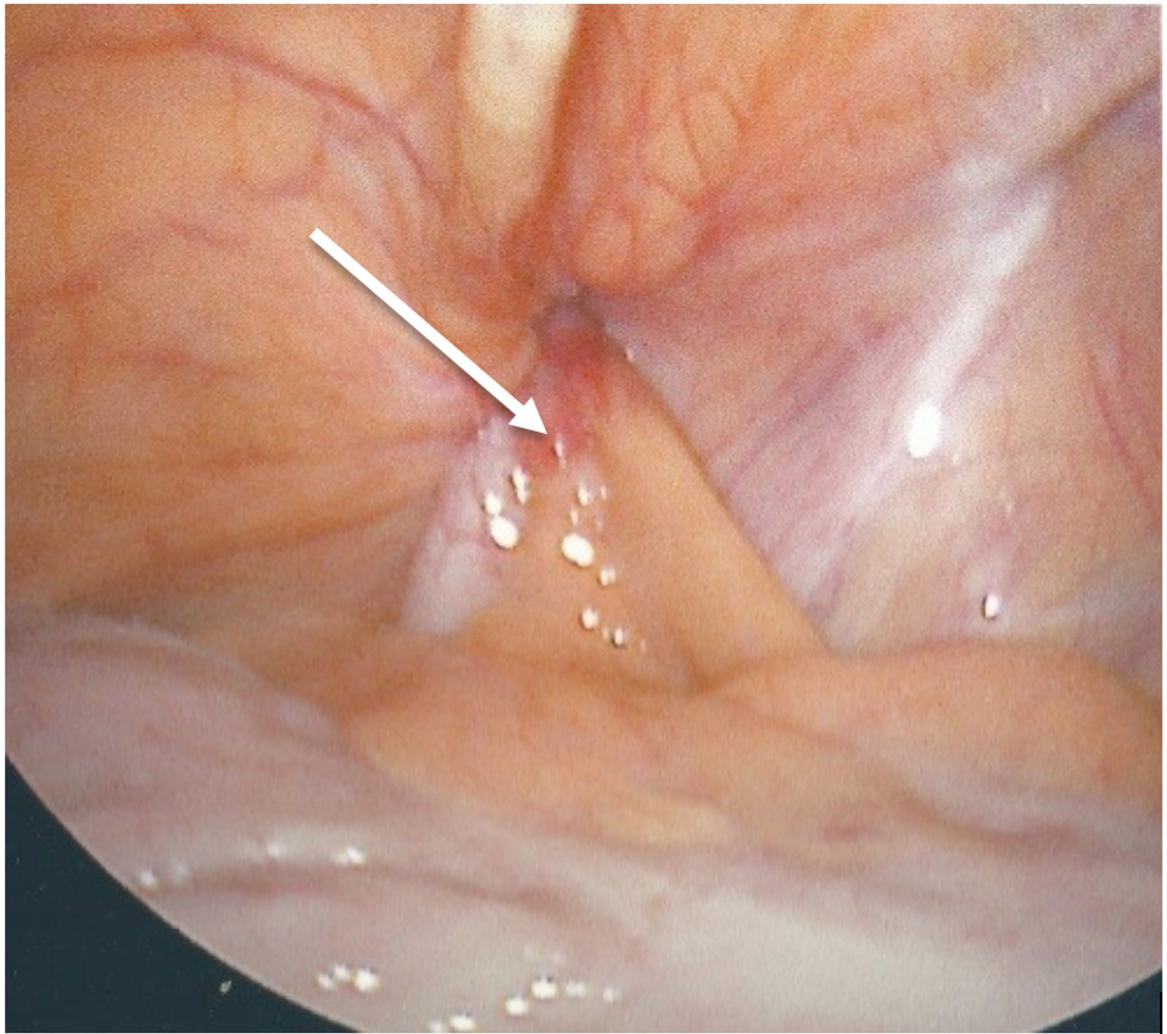
Laparoscopic view of inflamed appendix in right femoral hernia

She underwent laparoscopic exploration where an inflamed appendix entering the femoral hernia sac was found intraoperatively (Figure 2).

After reducing the appendix, an appendectomy was done, with thorough saline irrigation of the dependent areas. The hernial sac was reduced, and no mesh was placed. The postoperative course was uneventful as the patient tolerated a regular diet on postoperative day one and was discharged home. Three months follow up, the patient did not have a symptomatic inguinal hernia. 

Case two 

**Figure 3:**
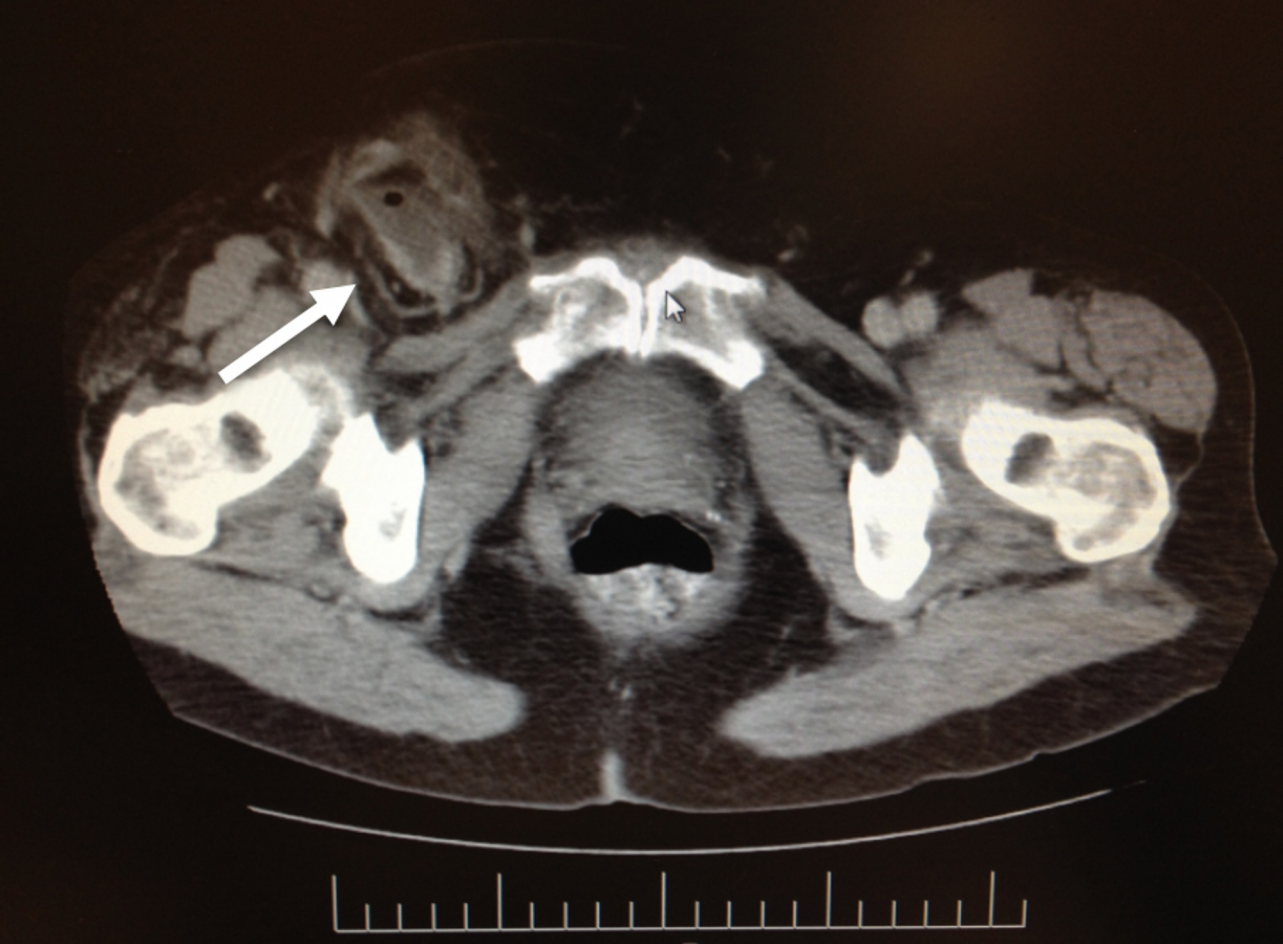
A CT scan demostrating the appendix herniating into right inguinal hernia with inflammatory changes surrounding it CT = computed tomography

A 56-year-old male with a past medical history significant for non-insulin-dependent diabetes mellitus presented with a painful right groin bulge. On arrival to the emergency room, the patient had sinus tachycardia with a heart rate of a 110 bpm. Physical examination was remarkable for pain and tenderness to palpation on right inguinal hernia. Blood work revealed leukocytosis of 21x10^3/mL. A CT scan of the abdomen and pelvis showed an incarcerated right groin hernia with an inflamed appendix as one of its contents (Figure 3).

**Figure 4:**
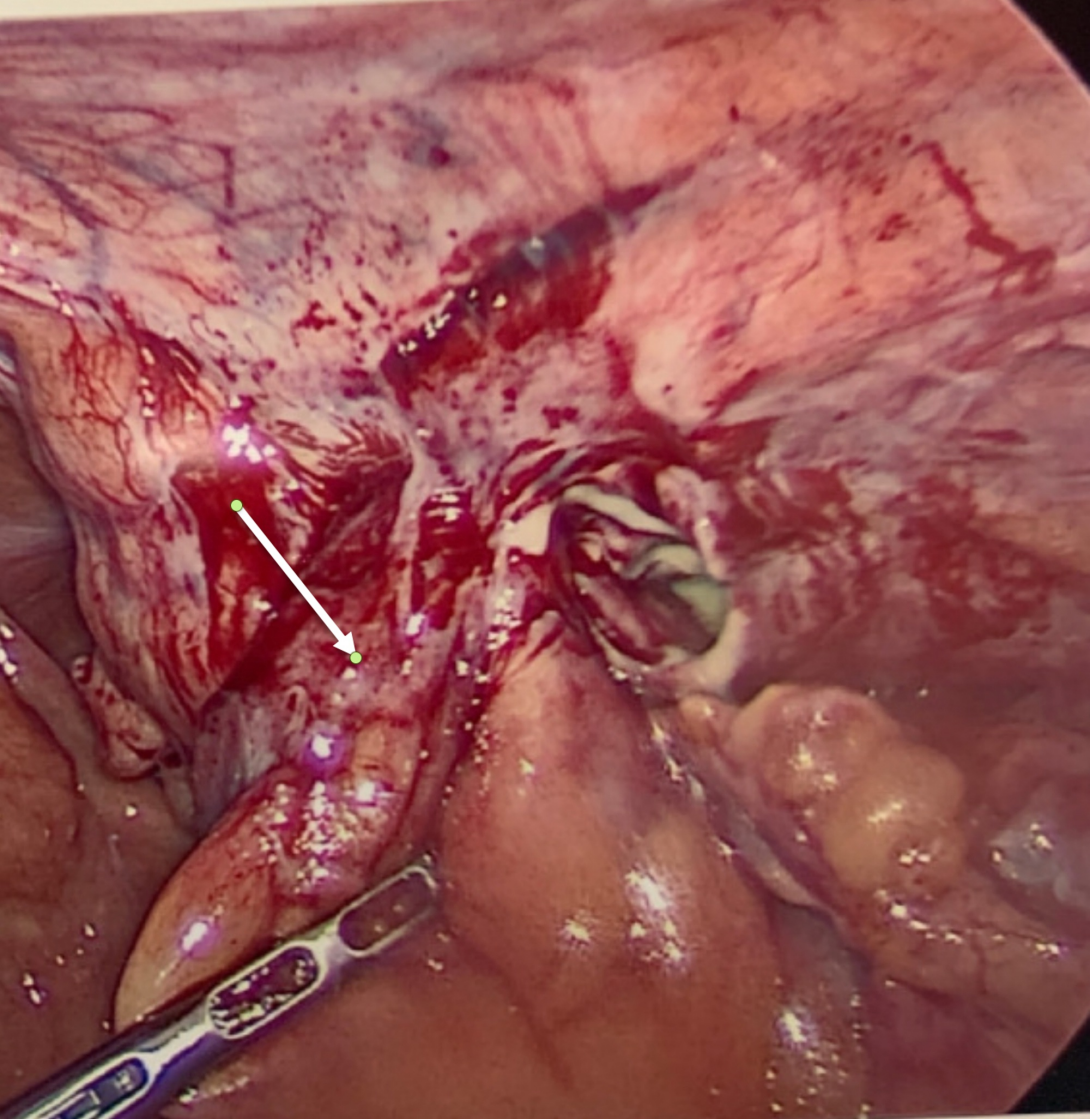
Purulent appendicitis in the hernial sac with extensive surrounding inflammation

The patient was taken emergently for laparoscopic surgery which revealed an inflamed and purulent appendix (Figure 4).

Since it was not possible to proceed with the reduction of the hernial sac laparoscopically in view of severe inflammation, conversion to open surgery occurred. First, the inflamed contents were mobilized laparoscopically and brought out via a small midline incision using a wound protector device. Second, an ileocolectomy and a side-to-side anastomosis was performed extracorporeally. Third, the contents were subsequently reduced back into the abdomen with the closure of the fascia using running polydioxanone (PDS) sutures. The postoperative course was complicated by a scrotal abscess that requiring drainage. The patient was discharged home on postoperative day seven with a one-week course of cephalosporin for *Escherichia coli* growth in wound. Three months follow up time was significant for no visible hernia.

Case three

**Figure 5:**
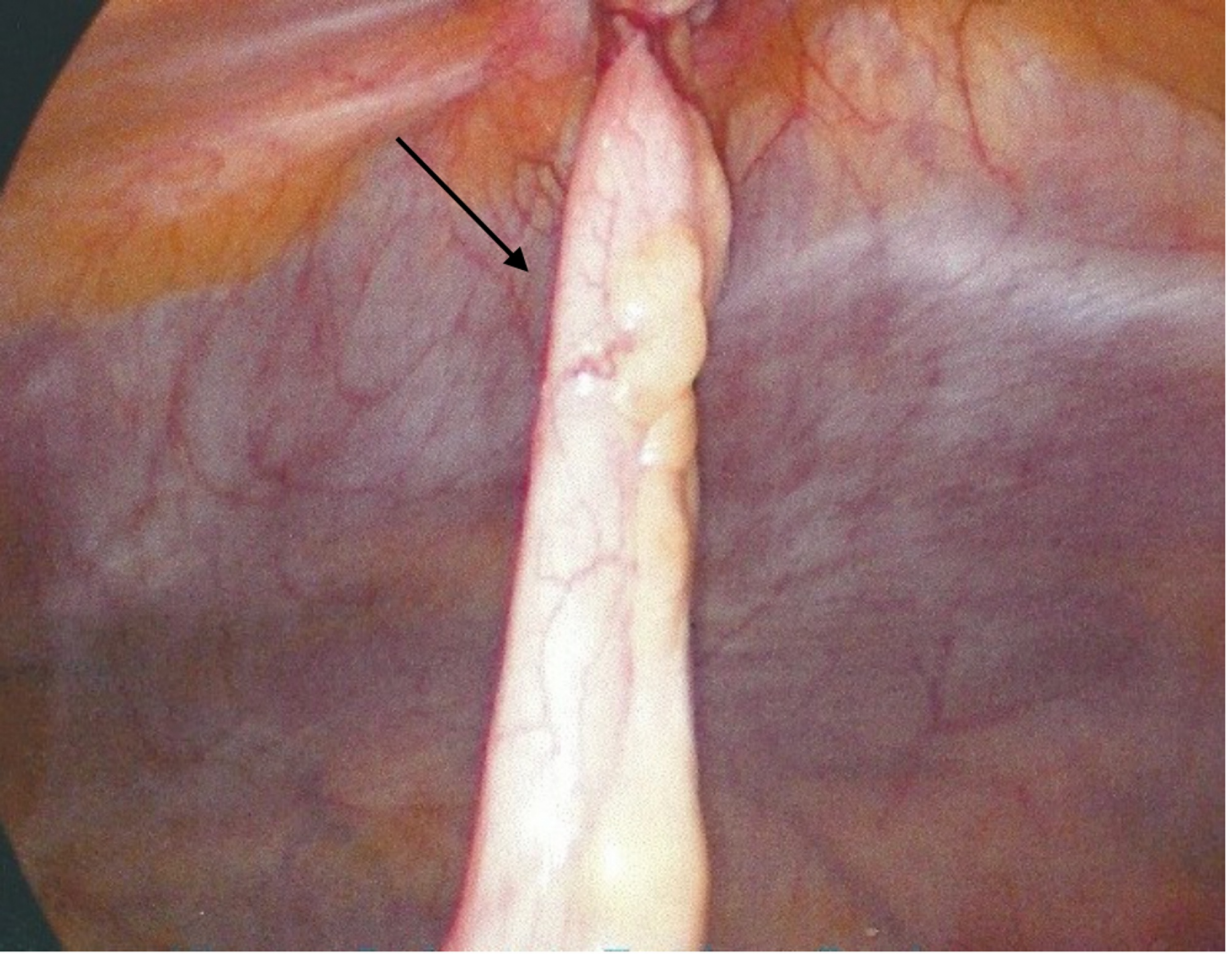
Laparoscopic view of inflamed appendix incarcerated in an umbilical hernia

A 45-year-old male patient with no past medical history presented with an acutely painful umbilical bulge. On arrival to the emergency room, his vital signs were stable and physical examination was positive for a very tender and erythematous bulge at the umbilicus consistent with an incarcerated hernia. Blood work was remarkable for leukocytosis to 11x10^3/mL. The patient was emergently taken for diagnostic laparoscopy and an inflamed appendix was seen incarcerated into the umbilical hernia (Figure 5).

An appendectomy was performed with saline irrigation of the pelvis and paracolic gutters after which no pockets of pus were seen. Primary repair of the hernial sac was performed with absorbable polyglactin sutures and the patient was discharged on postoperative day three. No visible hernia was seen on three months follow-up.

## Discussion

Rene Jacques Croissant de Garengeot, a French surgeon, was the first to describe the presence of the appendix inside an incarcerated femoral hernia in 1731 [4]. As previously mentioned, this type of hernia is rare in that it has fewer than 100 cases reported in the literature with its incidence varying between 0.5% and 5% of all femoral hernias [5]. Patients may present with fever, signs and symptoms that suggest an acute abdomen caused by obstruction, and laboratory studies may show non-specific results [6]. It occurs most frequently on the right side and there is a predisposition for females (13:1 women-to-men ratio) with this prevalence being attributed to body changes during pregnancy as well as other risk factors like increased intra-abdominal pressure, advanced age, smoking, and collagen defects [7]. 

In Amyand's hernia, the incidence of a normal appendix being found inside an inguinal hernia sac is about 1%, but it is inflamed in only about 0.1% of these cases [8]. It is usually on the right side due to the normal anatomical position of the appendix and there has been a classification system proposed by Lossanoff and Basson to guide treatment. This classification system comprises four distinct types based on the presence of increasing inflammation, to perforation and extra-abdominal pathology [9]. Patients typically present with a tender irreducible mass in the inguinal region, but the diagnosis is not always clear as it can be confused with a strangulated hernia [10]. CT imaging of both de Garengeot and Amyand's hernias may show air in the wall of an incarcerated hernia sac indicating intestinal involvement, but lack obstruction or dilatation of the small bowel [11]. 

The first adult case of appendicitis within an umbilical hernia was reported by Doig in 1970 [12]. There are no typical presentations and the variety of signs and symptoms has been described as ranging from nausea, vomiting, abdominal distension, and an erythematous, painful umbilical swelling consistent with a strangulated hernia to abscess formation with concurrent pain in the right lower quadrant [13]. CT findings for this phenomenon have been reported as a hernial defect containing the appendix and bowel with intramural air density and possible abscesses, but with no signs of obstruction or distension [3,13]. 

Treatment for acute appendicitis is appendectomy as conservative management non-operatively with antibiotics was inconclusive and should only be used for select patients were surgery is contraindicated [14]. Treatment for an incarcerated hernia is emergency hernia repair, with or without use of a mesh [15]. Typical treatment for de Garengeot's hernia has not been established but may consist of appendectomy followed by hernia correction, laparotomy for appendectomy and hernia correction by inguinotomy, or appendectomy through the hernia sac with correction of femoral hernia at the same time [16]. Treatment for Amyand's hernia is appendectomy with primary hernia repair. The use of synthetic mesh is avoided in the repair of abdominal defects if there is sign of infection due the risk of the prosthetic material increasing inflammation and possibly resulting in wound infection with the additional risk of an appendiceal stump fistula [8]. For an umbilical hernia with appendicitis, appendectomy and anatomical repair is the appropriate treatment as well [17]. Generally, laparoscopic hernia repair is contraindicated in patients with a history of lower abdominal surgery due to adhesions, abdominal radiotherapy, increased bleeding tendency, or in patients with a giant irreducible hernia [18]. The advantages of the minimally invasive technique are that there are low recurrence rates and it is associated with substantially less pain in the immediate postoperative period with earlier return to normal activities compared to open repair as was seen with our patients being out of the hospital within a week at the most after surgical intervention [19]. Finally, minimally invasive technique in inflamed hernias allows for easier approach to the base of the appendix for firing the stapler. In situations when it was not feasible to accomplish the surgery completely laparoscopically, it was possible to mobilize the contents, and thereby reduce the extent of abdominal incision needed to complete the surgery.

## Conclusions

In a small subset of patients where there is an incarcerated and inflamed appendix in the hernial sac, laparoscopy can provide comprehensive source control along with appendectomy and washout of dependent areas. Additionally, it also avoids a large incision, thereby permitting faster recovery. However, there may be limitation in feasibility of laparoscopy due to the individual surgeon's capability in this approach and the rarity of the situation. 
